# Variation in nest survival of three species of tropical plovers in Madagascar with clutch size, age of nest, year and El Niño effect

**DOI:** 10.1002/ece3.70269

**Published:** 2024-09-16

**Authors:** Claire E. Tanner, William Jones, Vojtěch Kubelka, Barbara A. Caspers, Oliver Krueger, Tafita Jaona Mijoro, Brett K. Sandercock, Sama Zefania, Tamás Székely

**Affiliations:** ^1^ Department of Biology and Biochemistry, Milner Centre for Evolution University of Bath Bath UK; ^2^ Faculty of Computing Engineering and Science, The University of South Wales Pontypridd UK; ^3^ Department of Evolutionary Zoology and Human Biology University of Debrecen Debrecen Hungary; ^4^ Department of Zoology and Centre for Polar Ecology University of South Bohemia České Budějovice Czech Republic; ^5^ Department of Behavioral Ecology Bielefeld University Bielefeld Germany; ^6^ Department of Animal Behaviour Bielefeld University Bielefeld Germany; ^7^ Faculty of Sciences University of Toliara Toliara Madagascar; ^8^ Department of Terrestrial Ecology Norwegian Institute for Nature Research Trondheim Norway; ^9^ Institut d'enseignement Supérieur de Menabe University of Toliara Morondava Madagascar

**Keywords:** clutch size, incubation, Madagascar, nest success, nest survival, reproductive strategies, shorebird, wader

## Abstract

A combination of life history traits and environmental conditions has been highlighted as the main drivers of avian breeding success. While drivers of breeding success are well known in some species, especially birds in northern, temperate regions; species in other parts of the world have received relatively little attention. In this study, we used a long‐term dataset on breeding success of tropical plovers from south‐west Madagascar to investigate whether nest survival changed over time and whether the drivers of nest survival were similar for multiple species breeding in the same arid habitat. In the 12‐year period of 2009–2020, we monitored 2077 nests for three species with different breeding strategies: 1185 nests of Kittlitz's plovers (*Anarhynchus pecuarius*) with a flexible breeding strategy and uniparental care; and 565 nests of white‐fronted plovers (*A. marginatus*) and 327 nests of Madagascar plovers (*A. thoracicus*) which both have biparental care. We found that nest survival was associated with a combination of clutch‐size, age of nest and year among the three plover species. In addition, annual variation in climatic conditions associated with El Niño/La Niña events were included in the most supported survival models for Kittlitz's and white‐fronted plovers, but the effects were not significant. Overall estimates of daily nest survival were similar for all three species: Kittlitz's plover: 0.950 ± 0.002 SE, Madagascar plover: 0.919 ± 0.007 SE, and white‐fronted plover: 0.862 ± 0.047 SE. Estimates of nest success for the breeding season, based on increases in daily nest survival with the clutch age during the incubation periods (26 days for Kittlitz's plovers and 29 days for Madagascar and white‐fronted plovers), were relatively low: Kittlitz's plover: 0.161 ± 0.056 SE, Madagascar plover: 0.287 ± 0.022 SE, and white‐fronted plover: 0.228 ± 0.019 SE. All three species had a combination of factors affecting nest survival, both environmental and life history traits.

## Graphical abstract

We found that nest survival was positively associated with a combination of clutch‐size and age of nest, in varying degrees, for the three plover species. Overall estimates of daily nest survival and nest success for the incubation period were similar for all three species. All three species had a combination of factors affecting nest survival, and nest survival was more affected by environmental or other extrinsic factors than variation in breeding strategy among the species.

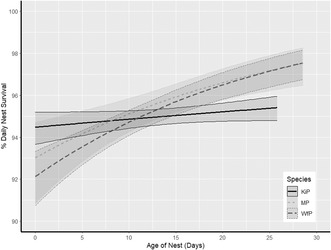

## 1 INTRODUCTION

Tropical and sub‐tropical bird species are relatively understudied when compared to their temperate and arctic counterparts (Xiao et al., 2017) and may have different factors affecting breeding success than temperate and arctic species (Roper et al., 2010). Tropical ecosystems differ from temperate and arctic ecosystems with climates more dependent on rainfall instead of temperature (Boyle et al., 2020) and are more strongly affected by quasi‐cyclical climatic phenomena, such as El Niño and La Niña events. Understanding the role of environmental factors that are associated with nest success is essential for understanding species' breeding systems, as declining reproductive success can create potential habitat sinks (Anteau et al., 2012).

Nest success varies among years and within seasons in most bird species, with the main environmental factors affecting nest success, including predation risk, climatic conditions, and food availability (Kwon et al., 2018; Smith et al., 2012). For most species, predation is the main cause of nest failure (Ellis et al., 2015; Kwon et al., 2018; Martin, 1993; Thompson, 2007). A decrease in nest survival can highlight high or increasing predation (Parr, 1992), from which conservation actions can be suggested (Roodbergen et al., 2012), such as predator control or nest exclusion devices (Dinsmore et al., 2014). However, studies have shown that predation risk is not always directly correlated with environmental factors, rather, it may be linked to the seasonal availability of other prey species in the same area (Smith & Wilson, 2010; Sperry et al., 2008). Losses to nest abandonment may increase seasonally if breeding birds encounter trade‐offs with timing of migration, moult or other energy‐demanding activities (Weiser et al., 2018). Thus, nesting date is also highly important in determining fate as it is often negatively correlated with nest survival (Claassen et al., 2014; Kosztolányi et al., 2009; Reneerkens et al., 2016).

Long‐term changes in climate and precipitation patterns have led to changes in habitat availability and quality (IPCC, 2021), which can negatively impact nest survival as egg‐laying species typically have a narrow range of temperatures which are optimal for development of the embryos (Nakage et al., 2003; Tanner et al., 2019). Climate change generally increases average temperatures and temperature variation, hence species living in tropical and sub‐tropical habitats may be more heavily affected because resident species experience relatively stable conditions, albeit with a narrower range in conditions (Sauve et al., 2021; Şekercioğlu et al., 2012).

In some species, seasonal changes in nest survival can be due to switches in sex‐specific parental care investment, which has been shown in species such as the Kentish plover (*Anarhynchus alexandrinus*) (Kosztolányi et al., 2009), and variation in individual strategies can occur both within populations and between populations. Despite a variety of parental care strategies being recorded in Al Wathba wetland reserve in UAE, biparental care was more common in this desert population than in other temperate populations (Kosztolányi et al., 2009). In addition, some species may reduce overall breeding activity when food availability is low (Engel et al., 2023), which can be due to seasonal or annual variation in climatic conditions.

It is important to increase our knowledge of equatorial habitats and species, as tropical animals may display different reactions to climatic changes than temperate species. For example, predation rates have been shown to be different between temperate and tropical shorebird species (Kubelka et al., 2018; Roper et al., 2010) and the differences have impacted selection (Roper et al., 2010). Roper et al. (2010) suggested that selection favours a short renesting interval where breeding seasons are long and predation rates high (i.e. tropical birds), whereas selection favours reduced reproductive rates where breeding seasons are short and predation rates are low (i.e., temperate birds). Environmental changes in predation rate could impact the future selection criteria.

Clutch size is one of the key variables potentially influenced by climate change or increasing predation pressure. Clutch size has previously been positively linked to hatching success in shorebirds (Larsen et al., 2003; Lengyel et al., 2009). Large clutches were shown to have significantly higher daily survival rates and hatching success in Northern lapwings (*Vanellus vanellus*) (Larsen et al., 2003). However, despite predation of nests being one of the main causes of nest failure in shorebirds (Kubelka et al., 2018), predation did not significantly differ with clutch sizes, suggesting that small clutches failed for reasons other than predation. In addition, clutch size and nest survival decreased in piping plovers (*Anarhynchus melodus*) with later nest initiation (Claassen et al., 2014), suggesting a link between the environmental factors decreasing nest survival and more intrinsic traits influencing clutch size.

Nest age also has the potential to affect nest survival in birds through changes in parental behaviour, such as nest defence (Larsen et al., 1996) or distraction displays (Smith & Wilson, 2010), or through seasonal changes in predation risk (Toral & Figuerola, 2012). Changes in daily nest survival with nest age have been reported in several shorebird species, including snowy plovers (*Anarhynchus nivosus*) (Claassen et al., 2014; Ellis et al., 2015; Hood & Dinsmore, 2007), upland sandpipers (Sandercock et al., 2015) and black‐winged stilts (*Himantopus himantopus*) (Toral & Figuerola, 2012). More vulnerable, or more conspicuous nests in ground‐nesting species may be depredated earlier in the incubation period creating a higher predation rate at the beginning of the incubation stage (Toral & Figuerola, 2012). Alternatively, species with biparental incubation may defend older nests more intensely (Smith & Wilson, 2010), which could increase the daily nest survival of older nests.

Lastly, mating systems and parental care are also linked to reproductive success (Székely et al., 2023). Nest survival is expected to be lower for nests with uniparental care, due to reduced nest attentiveness and increased activity in incubation breaks (Carroll et al., 2018; Meyers et al., 2019; Ricklefs et al., 2017). The costs associated with the decision to desert the partner and offspring or conversely to stay and provide parental care, include reproductive fitness, energy, time, and resources (Barta et al., 2002; Halimubieke et al., 2019, 2020; Owens & Bennett, 1997).

Madagascar provides an unusual opportunity to compare and investigate nest success in three congeneric species of plover, including Madagascar plover (*Anarhynchus thoracicus*), Kittlitz's plover (*A. pecuarius*) and white‐fronted plover (*A. marginatus*). The breeding distributions of the three species overlap within the same habitats (Zefania & Székely, 2013). The Kittlitz's plover is a common species across Africa (BirdLife International, 2016a). In contrast, the white‐fronted plover has an endemic subspecies (*A. m. tenellus*) in Madagascar, which is genetically and morphologically distinct from mainland African populations (Dos Remedios et al., 2020; Eberhart‐Phillips et al., 2015). Overall, the population trend for white‐fronted plovers is decreasing but the species is classified as least concern, in part because specific subspecies population trends are currently unknown (BirdLife International, 2016b). The Madagascar plover is a declining endemic species that is classified as globally vulnerable (BirdLife International, 2016c).

The three species of plovers share similar life histories and behaviour, as insectivorous ground nesting shorebirds with a modal clutch size of 2 or 3 eggs (Zefania & Székely 2022). However, they differ in their social systems since the Madagascar and white‐fronted plovers both display biparental care in incubation (Parra et al., 2014; Zefania & Székely, 2013), whereas the Kittlitz's plover's incubation care varies and can be either uniparental or biparental, with exact role distinctions varying among populations (Cunningham et al., 2018; Székely, 2019). For the majority of nests in Madagascar, one parent provides incubational parental care; however, the precise roles are unclear with some pairs potentially displaying incubation splits where one parent forages while the other incubates (Zefania & Székely, 2013). Putative nest predators in Madagascar include feral dogs, crabs, egg‐eating snakes, corvids, tenrecs and mongooses (Zefania & Székely, 2013).

Madagascar has two distinct wet and dry seasons, with high inter‐annual variation caused by the Southern Oscillation (ENSO), producing cycles of El Niño and La Niña events (Ropelewski & Halpert, 1987). In Madagascar, El Niño events typically result in warmer and drier‐than‐average conditions, while La Niña events result in cooler and wetter‐than‐average conditions (Randriamahefasoa & Reason, 2017). The climatic cycles are becoming more unpredictable, causing droughts to become longer and more intense, a pattern that is expected to intensify in the coming decades (Abiodun et al., 2019; Masih et al., 2014). Given the high levels of endemism in Madagascar, climatic effects could be catastrophic for the island's biodiversity (Antonelli et al., 2022; de Witt, 2003; Ralimanana et al., 2022). Certain habitats, such as the wetland habitats that plovers generally reside in, are disproportionately affected by climatic variability compared to other habitats (Shokoufeh et al., 2021). The three plover species in this study appear to have some flexibility in their timing and reproductive investment in breeding events, with many individuals skipping breeding during drought years (Jones et al., 2022).

In the context of a highly variable environment such as those with El Niño events, we assessed the survival of nests in these three plover species in Madagascar and the potential drivers. (1) Tropical habitats have a diverse predator community and a variable climate due to ENSO, so we predicted a lower nest survival overall for the three species compared to other shorebird species (Que et al., 2015). (2) A larger clutch size represents an increased investment of energy in a precocial species, such that pairs with large clutches may also invest more in incubation constancy and have higher nest survival. (3) Older nests are predicted to have a higher reproductive value and some bird species have been shown to increase nest defence with increased nest age. The “reproductive value hypothesis” describes a parental response to developmental changes (with offspring age) which result in a higher chance of survival for the offspring (Patterson et al., 1980; Williams, 1966). Thus, we predicted that nest survival should increase with nest age. (4) Uniparental nests generally have a lower nest success than biparental nests due to the lower rates of nest attendance in uniparental incubation. Thus, Kittlitz's plovers were predicted to have the lowest nest success out of the three species studied. (5) Nests incubated during El Niño and La Niña events are expected to have a lower nest survival than nests incubated during “normal” conditions. Here, we present some of the first demographic estimates of nest survival for three tropical species of plover, with a large dataset spanning 12 years. Our estimates of nest survival are among the first demographic data for any population of shorebirds in Madagascar.

## 2 METHODS

### 2.1 Data collection

Field data were collected at the Andavadoaka area in south‐western Madagascar (22°04′ S, 43°14′ E) between 17th February and 14th August in the 12‐year period of 2009 to 2020 (inclusive). The field site consists of a series of ephemeral saltmarshes adjacent to the coast and was initially selected as a study site due to high numbers of breeding plovers (Long et al., 2008; Zefania et al., 2008). Nests were located by using a combination of different search techniques and monitored until completion (Székely et al., 2008). Nest age at the date of location was determined via the floatation method, which has been calibrated for each of these three species from known lay dates (Table S2). The nests were monitored at regular intervals (every 2 to 4 days) from the found date until they successfully hatched, or failed because they were depredated, trampled, flooded, or abandoned. If a nest was not attended by parents (i.e. parents were either on an incubational off‐bout or had abandoned), a twig was placed over the nest. If parents were present and the nest was active, the twig was removed from the nest by the parents before the next nest check.

The date the nest was first discovered by the observer was labelled as the “found date.” The “last active date” was recorded as the date of the last nest check in which the nest still had eggs present and was actively attended by the parents. The “date of completion” was recorded as either the date when at least one of the eggs successfully hatched, when the chicks were seen as hatched or alternatively the first date of inactivity for nests which failed or cases where the eggs were missing due to an unknown fate. In a few cases where the nest was not followed after completion of the fieldwork (9 Kittlitz's plover nests, 4 Madagascar plover nests and 16 white‐fronted plover nests), the date of completion was coded as the same as the last active date; the nest was considered to have “survived” for the study period but with an “unknown” fate. For the analyses with the nest survival model, the last active and date of completion were different and bracketed the date of loss for failed nests, whereas the two dates were set to be the same date for successful nests. Nests which had zero exposure because they were only visited once, and nests manipulated for experimental studies were not included in the analyses.

### 2.2 Nest fate

Nest fate was determined using a standardised protocol so that all observers were consistent in their methods for nest fate determination. A nest was considered successful if at least one of the eggs hatched, or if the nest was active on the last day that it was checked at the end of the field season. Nest were designated as failed if they were determined to have been depredated, flooded, trampled, or abandoned at the last visit (Table S1). A nest was labelled as: *hatched* if the chicks were caught at the nest or seen after hatching with a individually marked parent, *depredated* if there were signs of predation such as broken eggs, predator tracks or camera footage of predation; *abandoned* if a twig was placed over the nest and not removed by a parent after three consecutive nest checks; and *other* for flooded or trampled. For abandoned nests, the last active date was recorded as the last check date before a twig was placed over the nest and the date of completion was the date the twig was placed. Hatched nests were considered successful, whereas depredated, abandoned, flooded and trampled nests were considered failed. Nests with infertile eggs were removed from the analysis (4 nests of Kittlitz's plover), as they were neither destroyed by depredation, flooding or trampling nor did the eggs hatch. If the nest was either not followed until hatching, or if the nest could not be labelled as failed or hatched, it was recorded as unknown fate.

For nests with unknown fates where eggs disappeared from the nest scrape, the hatching date was estimated using calibrations of known laid dates against floatation data (see Table S2 for details) and compared to the last date recorded. The nest was considered “failed” if the midpoint date halfway between the last active and the date of completion was before the estimated hatching date. Conversely, the nest was classified as “successful” if the midpoint was after the estimated hatching date.

All dates were recorded in days of the year (1 to 365) with day 1 set as the earliest date that the first nest was found in the breeding season. Day 1 is not set as January due to the seasonal nature of the species' breeding. “Time in season” is the full dependence throughout the breeding season.

### 2.3 Statistical analyses

All analyses were conducted with the nest survival model in program MARK (White & Burnham, 1999), and daily survival rates of nests (DSR) were modelled separately for each of the three species of plovers. Encounter histories for each nest included date found, last active date, date of completion, and nest fate (0 = successful, 1 = failure), and were grouped by year, with clutch size, age of nest and El Niño/ La Niña included as individual covariates. All models tested are reported in Tables 1, 2, 3. Clutch size was calculated as the maximum clutch size recorded. Age of nest was coded as the estimated age of the nest on day 1 of the nesting season with negative values for dates before the clutches were initiated. Data on El Niño/ La Niña events was taken from the National Oceanic and Atmospheric Administration website (Climate Prediction Center Internet Team, 2024). The covariate was coded as 1 for nests incubated within El Niño conditions, 2 for nests incubated within normal conditions and 3 for nests incubated within La Niña conditions. El Niño events occurred July 2009 to March 2010, October 2014 to April 2016 and September 2018 to June 2019. La Niña events occurred June 2010 to April 2012, August 2016 to December 2016, October 2017 to April 208 and April 2020 until the end of the study period. If nests were partially incubated in multiple conditions, the condition in which the nest was incubated in for the majority of the time was used. All records with missing information on clutch size and age of nest were discarded for these models (21 nests total). Akaike's Information Criterion corrected for small sample sizes (AICc) was used to rank alternate models (Akaike, 1973). The parameter counts were taken from the MARK output and could be less than the full model structure. For a few years of groups with sparse data, daily nest survival could not be estimated.

**TABLE 1:** Models of daily survival rates for nests of Kittlitz's plovers (*n* = 1185) in Madagascar, 2009–2010 and 2012–2020. Model factors included year (11 years), clutch size (1 vs. 2 eggs), age of nest (1–25 days), and the El Niño/La Niña effect (period of El Niño, period of La Niña and normal period). “Time” is the full time‐dependence throughout the breeding season. Bold values are those with AICc weights of over 0.

Model	AICc	Delta AICc	AICc weights	Num. Par (*K*)
Year × (Age of nest + Clutch size)	3621.55	0.00	**0.41**	32
Clutch size	3622.64	1.09	**0.24**	2
Age of nest + Clutch size	3623.46	1.91	**0.16**	3
Age of nest + Clutch size + El Niño/La Niña	3624.16	2.61	**0.10**	4
Clutch size + El Niño/La Niña	3624.95	3.40	**0.08**	4
Year × Age of nest	3649.30	27.75	0.00	18
Year	3650.18	28.63	0.00	11
Age of nest	3654.41	32.86	0.00	2
Age of nest + El Niño/La Niña	3654.53	32.98	0.00	3
Intercept‐only	3655.61	34.06	0.00	1
El Niño/La Niña	3654.88	35.33	0.00	3
Year × Clutch size	3695.00	73.45	0.00	20
Time	3768.68	147.13	0.00	123

**TABLE 2:** Daily survival of Madagascar plover nests in relation to year, clutch size, age of nest and El Niño/La Niña (*n* = 327 nests), in Madagascar, 2009–2010, and 2013–2019. Model factors included year (9 years), clutch size (1 vs. 2 eggs), age of nest (1–28 days), and the El Niño/La Niña effect (period of El Niño, period of La Niña and normal period). “Time” is the full time‐dependence throughout the breeding season. Bold values are those with AICc weights of over 0.

Model	AICc	Delta AICc	AICc weights	Num. Par (*K*)
Year × (Age of nest + Clutch size)	854.87	0.00	**0.65**	23
Year × Clutch	856.15	1.28	**0.34**	15
Year × Age of nest	865.45	10.58	0.00	16
Clutch size	868.26	13.39	0.00	2
Year	868.57	13.70	0.00	8
Age of nest + Clutch size	870.25	15.38	0.00	3
Age of nest + Clutch size + El Niño/La Niña	871.92	17.05	0.00	4
Clutch size + El Niño/La Niña	871.93	17.05	0.00	4
Intercept‐only	873.75	18.88	0.00	1
Age of nest	875.70	20.83	0.00	2
El Niño/La Niña	879.16	24.29	0.00	3
Age of nest + El Niño/La Niña	879.16	24.29	0.00	4
Time	939.06	84.19	0.00	121

**TABLE 3:** Daily survival of white‐fronted plover nests in relation to year, clutch size, age of nest and El Niño/La Niña (*n* = 565 nests) in Madagascar, 2009–2020. Model factors included year (12 years), clutch size (1, 2 vs. 3 eggs), age of nest (1–28 days), and the El Niño/La Niña effect (period of El Niño, period of La Niña and normal period). “Time” is the full time‐dependence throughout the breeding season. Bold values are those with AICc weights of over 0.

Model	AICc	Delta AICc	AICc weights	Num. Par (*K*)
Year × (Age of nest + Clutch size)	1388.39	0.00	**0.91**	30
Year × Age of nest	1392.98	4.58	**0.09**	21
Age of nest	1405.31	16.91	0.00	2
Year × Clutch size	1418.46	30.07	0.00	20
Age of nest + Clutch size + El Niño/La Niña	1443.11	54.71	0.00	5
Age of nest + Clutch size	1442.17	54.77	0.00	4
Year	1449.19	60.79	0.00	12
Clutch size	1453.39	64.99	0.00	3
Intercept‐only	1469.44	81.05	0.00	1
El Niño/La Niña	1473.01	84.61	0.00	3
Clutch size + El Niño/La Niña	1533.69	145.01	0.00	5
Age of nest + El Niño/La Niña	1642.97	254.58	0.00	4
Time	1723.50	335.11	0.00	179

Daily nest survival estimates were extracted from the models for graphical visualisation. Following recommendations of Weiser (2021), nest success for the incubational period was estimated as the product of consecutive estimates using R (R Core Team, 2020). The product of consecutive estimates used daily nest survival estimates that were extracted from the 3‐factor model S (Year × (Age of nest + Clutch size)). This candidate model was the highest ranked model for all three species of plovers.

## 3 RESULTS

### 3.1 Nest monitoring

A total of 2077 nests from three species of tropical plovers were monitored at Andavadoaka, Madagascar in the 12‐year period of 2009 to 2020 (Table S1). However, the plovers sometimes skipped breeding in drought years; hence, no data could be collected on breeding or nest survival during 2011, 2012 and 2020 for some species (Figure S1). A majority of the nests were Kittlitz's plover (*n* = 1185) with fewer nests for Madagascar plovers (*n* = 327) and white‐fronted plovers (*n* = 565) (Table S1). Duration of incubation varied from 26–29 days among the three study species (Table S2). Most nests were found at the start of incubation in Kittlitz's and Madagascar plovers, but at different stages of the incubation period for white‐fronted plovers (Table S3). Overall, between ~29% of nests hatched in each species, with predation as the main cause of failure (~46%), followed by nest abandonment, flooding, and trampling. Nests of unknown fate accounted for ~17% of nests in each species (Table S1).

### 3.2 Life history variables and annual variation in daily Nest survival

**FIGURE 1:**
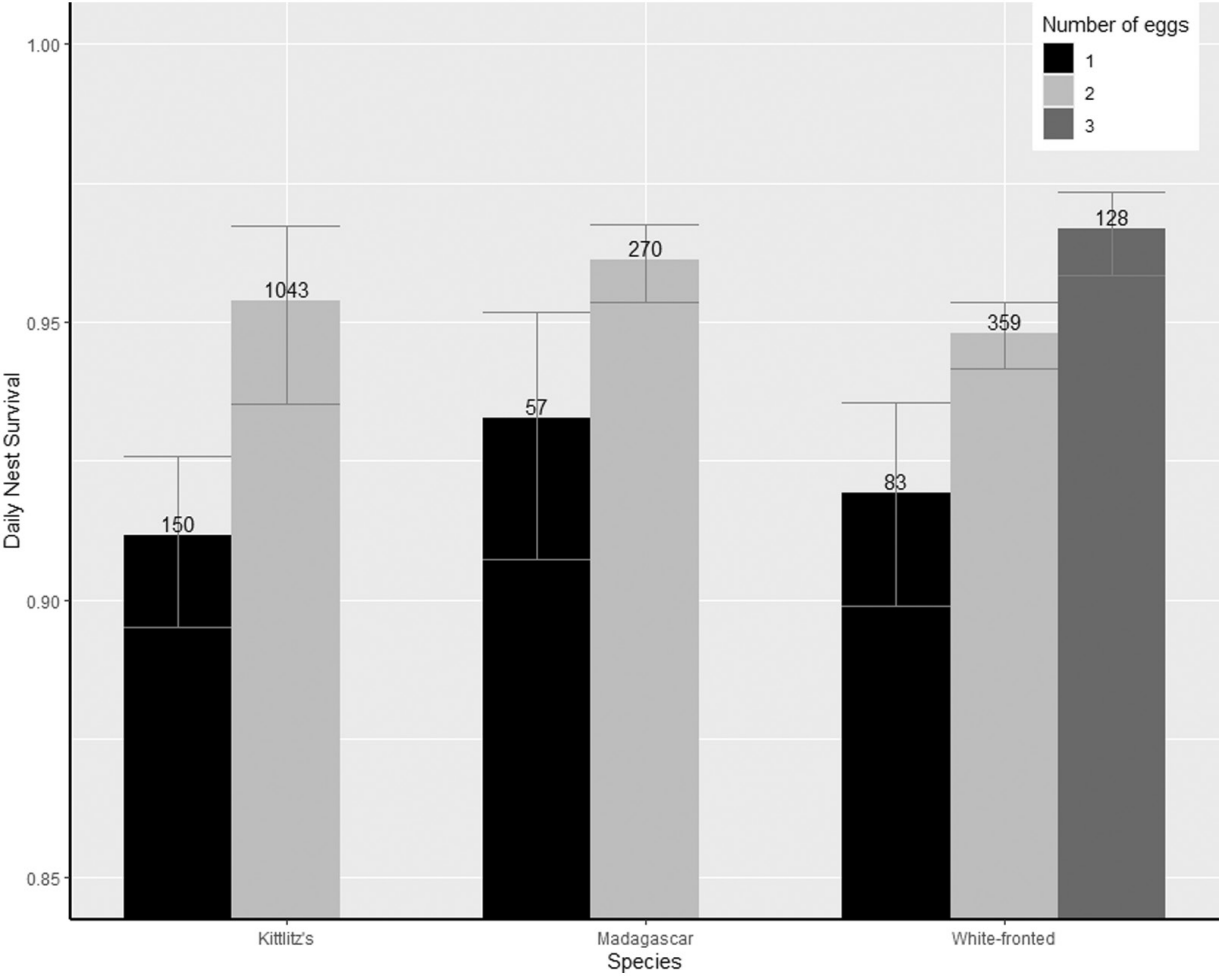
Probability of daily nest survival (95%CI, sample size of nests) in relation to clutch size for three species of tropical plovers in Madagascar. One egg clutches in black, 2 egg clutches in light grey and 3 egg clutches in dark grey. Parameter estimates taken from the “Clutch size” models.

**FIGURE 2:**
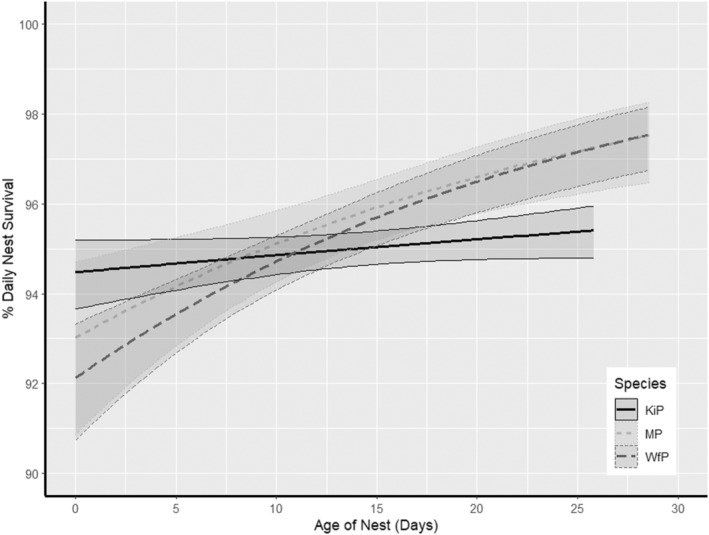
Estimated daily nest survival (95%CI) in relation to age of nest for three species of tropical plover in Madagascar. Kittlitz's plovers (KiP) in black with a solid line, Madagascar plovers (MP) in light grey with a short dashed line and white‐fronted plovers (WfP) in dark grey with a long dashed line. Parameter estimates taken from model (nest age).

**FIGURE 3:**
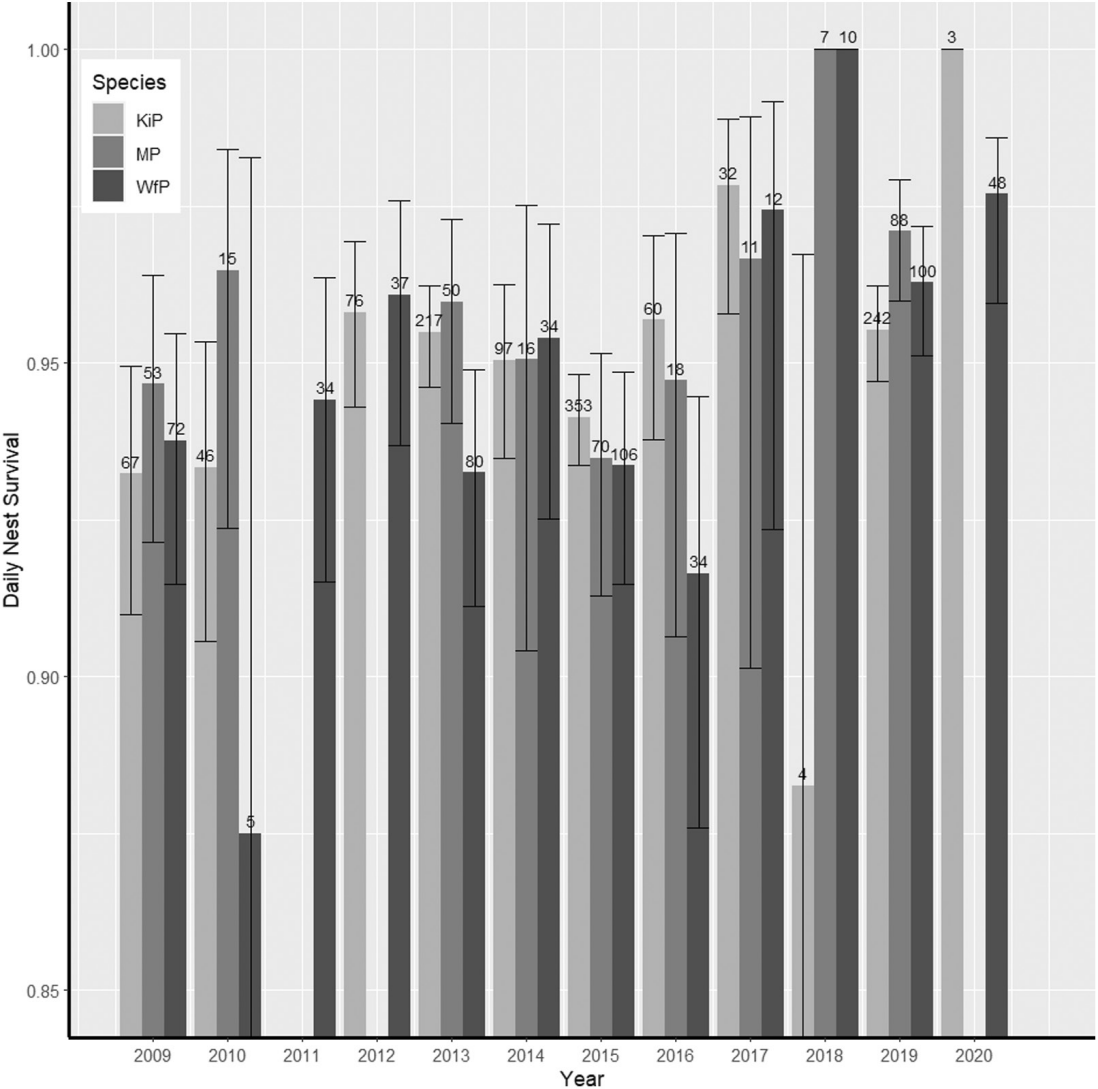
Daily nest survival (95%CI, sample size of nests) of Malagasy plovers over the 12‐year study period of 2009–2020. Kittlitz's plovers (KiP) in light grey, Madagascar plovers (MP) in mid grey and white‐fronted plovers (WfP) in dark grey. The missing estimates in 2011, 2012 and 2020 are years without nesting records for Kittlitz's and Madagascar plovers. For Kittliz's plovers, no nests were found during these years. For Madagascar plovers, nests for this species were not recorded in 2012 despite a low number of pairs breeding during this year. Daily nest survival estimates were taken from the “Year” model.

**FIGURE 4:**
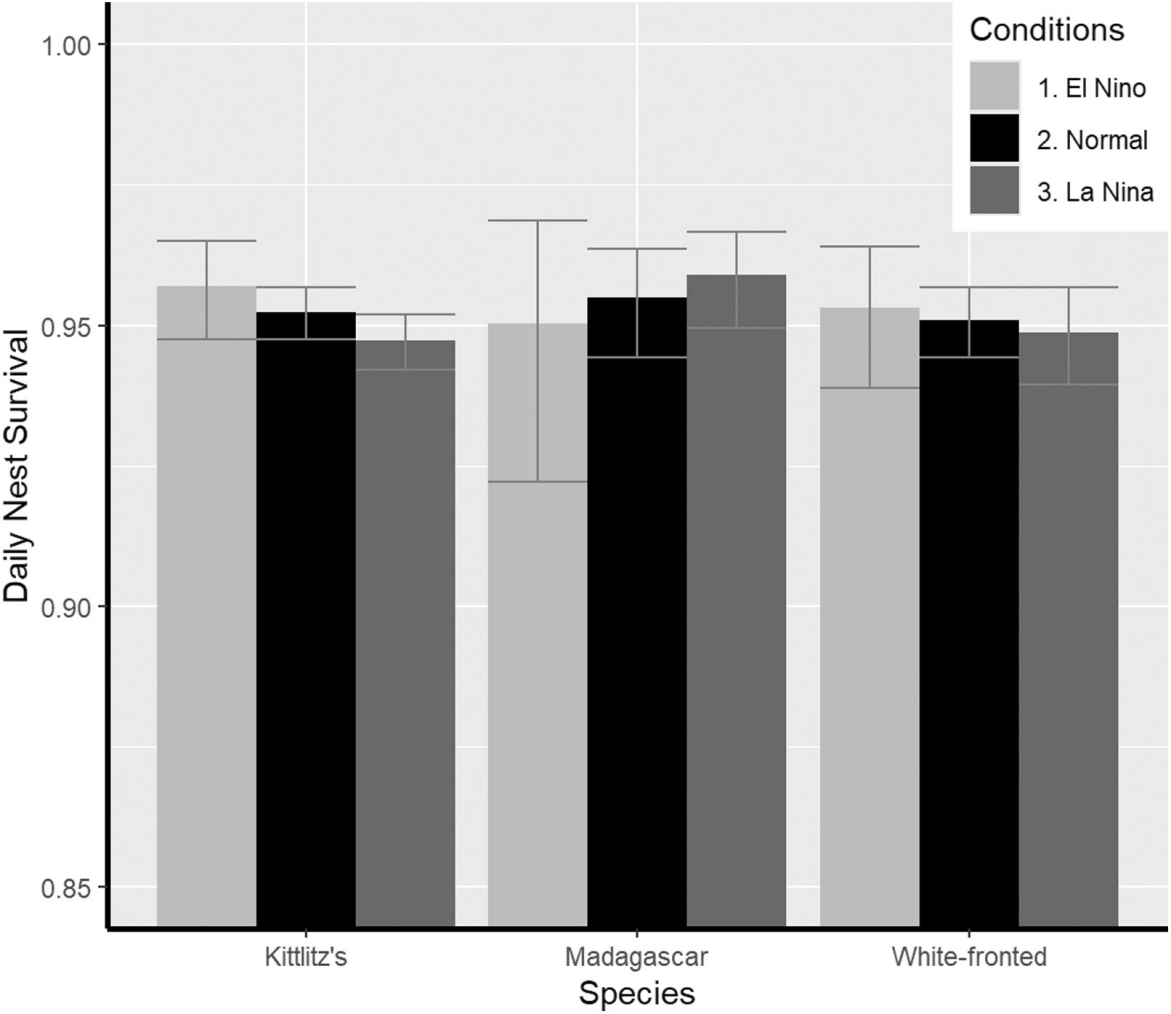
Probability of daily nest survival in relation to El Niño (a warm and dry climatic effect) or La Niña (a cool and wet climatic effect) events in each species with 95% CI. El Niño events in light grey, Normal events in black and La Niña in dark grey. Parameter estimates taken from model (El Niño/La Niña).

Clutch size, age of nest and year were the main factors affecting daily survival rates (DSR) of nests in all three species (Tables 1, 2, 3). Complete 3‐egg/2‐egg clutches (in white‐fronted plovers) and 2‐egg clutches (in Kittlitz's and Madagascar plovers) had a higher DSR than smaller clutches (Figure 1, Table 4). In addition, DSR increased with nest age, as predicted (Figure 2, Table 4). There was an effect of age of nest whereby daily nest survival rates increased with clutch age (Figure 2). The same pattern of increase was observed in all three species but was more pronounced in white‐fronted plover and Madagascar plover. Daily nest survival varied among years, but the overall long‐term trend did not increase nor decrease over the study period (Figure 3). Models with El Niño/ La Niña were only supported within Kittlitz's plovers, but were not in the top model for any species (Figure 4). However, climatic state was not a significant factor within the model in nest survival across climatic phenomena. Models with constant survival or full time‐dependence within the season (the “Intercept” and “Time”) received little support among the candidate models (Tables 1, 2, 3). Madagascar and white‐fronted plovers were more sensitive to the year (both with AICc Weight 1 for models with year and 0 for models without year), whereas white‐fronted plovers were more sensitive to the effect of age of nest than both of the other two species (White‐fronted plover AICc Weight 1 for age and 0 for models without age; Madagascar plover AICc Weight 0.66 for age and 0.34 for models without age; Kittlitz's plover AICc Weight 0.68 for age and 0.32 for models without age).

**TABLE 4:** Beta estimates for covariates for the top models for Kittlitz's, Madagascar and white‐fronted plovers. Factors which are not included in the final model for each species have been left blank. Beta estimates are presented on a logit scale.

Year	Factor parameter	Beta estimates ± standard error		
		Kittlitz's	Madagascar	White‐fronted
2009	Intercept	−0.55 ± 0.90	0.92 ± 0.75	0.42 ± 0.76
	Clutch size	1.58 ± 0.50	0.03 ± 0.02	−0.00 ± 0.02
	Age of nest	0.013 ± 0.02	0.92 ± 0.43	1.18 ± 0.43
	El Niño/La Niña	—	—	—
2010	Intercept	3.60 ± 0.87	4.70 ± 1.76	2306.15 ± 0.00
	Clutch size	−0.01 ± 0.02	0.03 ± 0.05	−29.00 ± 0.00
	Age of nest	−0.46 ± 0.46	−1.31 ± 0.89	−972.62 ± 0.00
	El Niño/La Niña	—	—	—
2011	Intercept	—	—	−0.02 ± 1.45
	Clutch size	—	—	0.01 ± 0.02
	Age of nest	—	—	1.33 ± 0.78
	El Niño/La Niña	—	—	—
2012	Intercept	−244.95 ± 0.00	—	0.44 ± 1.26
	Clutch size	−0.02 ± 0.01	—	0.04 ± 0.03
	Age of nest	124.23 ± 0.00	—	0.81 ± 0.54
	El Niño/La Niña	—	—	—
2013	Intercept	1.64 ± 0.44	1.34 ± 0.85	0.11 ± 0.49
	Clutch size	0.02 ± 0.01	0.06 ± 0.03	0.08 ± 0.04
	Age of nest	0.66 ± 0.23	0.59 ± 0.48	0.57 ± 0.26
	El Niño/La Niña	—	—	—
2014	Intercept	0.39 ± 0.86	4.21 ± 2.20	2.03 ± 1.23
	Clutch size	0.00 ± 0.02	−0.04 ± 0.05	0.09 ± 0.04
	Age of nest	1.32 ± 0.45	−0.22 ± 1.11	−0.34 ± 0.54
	El Niño/La Niña	—	—	—
2015	Intercept	1.21 ± 0.00	−2.43 ± 1.40	1.27 ± 0.54
	Clutch size	0.00 ± 0.01	−0.01 ± 0.02	0.07 ± 0.02
	Age of nest	0.83 ± 0.07	2.67 ± 0.75	0.19 ± 0.22
	El Niño/La Niña	—	—	—
2016	Intercept	4.07 ± 2.21	−1.88 ± 2.50	0.61 ± 1.19
	Clutch size	0.01 ± 0.02	−0.09 ± 0.06	0.03 ± 0.03
	Age of nest	−0.54 ± 1.06	2.96 ± 1.41	0.61 ± 0.55
	El Niño/La Niña	—	—	—
2017	Intercept	110.53 ± 0.00	2.74 ± 0.00	−838.72 ± 0.00
	Clutch size	−0.06 ± 0.03	−0.04 ± 0.08	0.06 ± 0.11
	Age of nest	−52.90 ± 0.00	0.61 ± 0.00	420.68 ± 0.00
	El Niño/La Niña	—	—	—
2018	Intercept	18.44 ± 7929.60	12.79 ± 0.00	845.54 ± −0.58E+10
	Clutch size	−2.39 ± 0.00	0.01 ± 0.00	0.29 ± 0.00
	Age of nest	9.22 ± 3946.80	2.83 ± 0.00	31.85 ± 0.17E+10
	El Niño/La Niña	—	—	—
2019	Intercept	1.69 ± 0.47	2.05 ± 0.85	1.89 ± 0.49
	Clutch size	0.01 ± 0.01	0.05 ± 0.02	0.06 ± 0.02
	Age of nest	0.67 ± 0.25	0.39 ± 0.45	0.11 ± 0.21
	El Niño/La Niña	—	—	—
2020	Intercept	7.74 ± 3937.87	—	2.88 ± 1.07
	Clutch size	0.01 ± 0.00	—	0.00 ± 0.02
	Age of nest	3.87 ± 1968.93	—	0.42 ± 0.50
	El Niño/La Niña	—	—	—

### 3.3 Overall estimates of Nest success

Daily nest survival was similar for the three species: Kittlitz's plover: 0.950 ± 0.002 SE, Madagascar plover: 0.919 ± 0.007 SE, white‐fronted plover: 0.862 ± 0.047 SE. The duration of the incubation period for Madagascar plovers (28.6 days ±0.3 SE, *n* = 139) and white‐fronted plovers (28.5 days ±2.3 SE, *n* = 100) was approximately 3  days longer than Kittlitz's plovers (25.8 days ±0.3 SE, *n* = 220). However, variation in exposure did not appear to affect reproductive output, as the Madagascar plover had a slightly higher nest success rate than the Kittlitz's and white‐fronted plovers. Estimates of incubational nest success based on the product of daily survival rates from the “Year × (Age of nest + Clutch size)” models were < 0.30 for all three species: Kittlitz's plover: 0.161 ± 0.056 SE, Madagascar plover: 0.287 ± 0.022 SE, and white‐fronted plover: 0.228 ± 0.019 SE.

## 4 DISCUSSION

Our long‐term research project provides the first estimates of nest survival for a group of poorly known and understudied community of tropical plovers in Madagascar. Nest success of the three tropical plovers was relatively low (<0.30) compared to the average nest success of Kentish or snowy plovers breeding in temperate habitats (mean 0.445, range = 0.168 to 0.840, Que et al., 2015). Important drivers included an effect of clutch size on daily nest survival, increases in daily nest survival with nest age, an effect of year on daily nest survival, a weak effect of El Niño/ La Niña on daily nest survival but relatively little difference in incubational nest survival among species with different parental care strategies. Different species did, however, exhibit differences in the sensitivity of life history traits and yearly differences. Madagascar and white‐fronted plovers were more sensitive to annual variation, and white‐fronted plovers were more sensitive to the effect of age of nest than both of the other two species. The interspecific differences were interesting because Kittlitz's plover is the species with the flexible parental care system and also the species that has arrived most recently on Madagascar (Eberhart‐Phillips et al., 2015). Below, we evaluate the potential mechanisms underlying each of the key drivers of the observed variation in nest survival.

Clutch size was one of the predictors included in the models with the highest likelihood, with larger complete clutches having a higher nest survival than smaller clutches. An increase in clutch size is generally linked to higher quality parents, which could be due to a better diet in high quality territories (Högstedt, 1980) or the coordinated incubational ability of mated pairs (Székely et al., 1994), with total nest attendance being higher in pairs with larger clutches (Larsen et al., 2003). Our results are contrary to previous experimental studies which found that larger clutches could reduce overall nest survival by 1.7% to 3.8% in shorebirds (Arnold, 1999) due to an increased incubation period (+3.2 days in Kentish plovers, Székely et al., 1994, +1.3 days in two calidridine sandpipers, Sandercock, 1997), which caused additional exposure time to predation risk. An alternative hypothesis for an effect of clutch size on nest survival could be due to life‐history trade‐offs. Trade‐offs in parental investment can include an individual lowering their survivorship to increase their reproductive output (Veasey et al., 2001). In experimental nests of Lesser Elaenia (*Elaenia chiriquensis*) with a larger clutch size, parents invested more, providing more parental care during both incubation and chick care (Sousa & Marini, 2013). Although there was no change in the length of incubation, post‐hatching growth of chicks was negatively affected by the increased clutch size. Alternatively, the survivorship trade‐off could be in response to the level of disease, which lowers the survival of adults or reduces the breeding probability in the following year (Descamps et al., 2009). Trade‐off strategies have also been proposed in Malagasy species to account for the comparatively long lifespans and apparent adult survival observed in the three species, with longevity estimates of 9.7 years (±1.1) in Kittlitz's plovers, 9.4 years (±1.0) in Madagascar plovers and 12.5 years (±1.3) in white‐fronted plovers (Jones et al., 2022). Plover populations in China with a higher adult survival rate contained fewer, larger eggs in a clutch, whereas populations with a lower adult survival rate produced larger clutches with smaller individual eggs (Song et al., 2020). However, Madagascar plovers do not have lower clutch sizes compared to Kittlitz's plovers, which suggests these species in Madagascar may not be displaying a survival trade‐off. Only two cases of partial depredation were recorded in our field study (both in Madagascar plovers in 2019) and nests were usually found soon after laying (Table S3). Thus, the reduced nest survival in one‐egg clutches was not likely to have been caused by partial predation.

Daily nest survival increased with the age of the nest in all three plover species and was included in the models with the highest likelihood. Our results for tropical plovers are similar to other temperate populations of shorebirds (Claassen et al., 2014; Ellis et al., 2015; Hood & Dinsmore, 2007; Sandercock et al., 2015; Toral & Figuerola, 2012). Increases in daily survival rates could be due to higher predation at the beginning of the incubation stage, for example the nests which are more vulnerable are depredated earlier in incubation (Toral & Figuerola, 2012). On the other hand, Smith and Wilson (2010) investigated adult shorebird behaviour during nesting and found that adults changed their behaviour during incubation and that some biparental species of shorebird increased nest defence closer to hatching. These changes in behaviour do not seem to affect the three species' nest survival in this study. Increased nest attendance by biparental couples would therefore provide more nest defence and more distraction displays, as there is a parent constantly attending the nest rather than periods of time where the adult is away from the nest. Uniparental species have a nest recess frequency three times higher and a total recess duration seven times higher than that of biparental species (Moreau et al., 2018). In addition, biparental parents significantly increase their nest survival if they display greater synchrony between timing of their individual recesses (Leniowski & Węgrzyn, 2018). However, common redshanks (*Tringa totanus*) displayed a reduced nest attendance when nesting in areas of high predation than in areas of low predation (Cervencl et al., 2011), which could potentially increase the predation of nests if the eggs are left unattended. However, such variation in predation risk is unlikely to affect the species in our study as the predation rates for these populations were not unusually high, compared to other shorebird species. A global comparative study of cause‐of‐failure showed current global total predation rates for shorebirds as an average of 57% ± 2% since 2000 (Kubelka et al., 2018), whereas the species in this study showed total predation rates of 44.1% ± 0.03% for Madagascar plovers, 44.4% ± 0.02% for white‐fronted plovers and 48.3% ± 0.02% for Kittlitz's plovers (Table S1).

Apparent nesting effort in all three species of plovers was variable among years in Madagascar (Figure S2) with some years with low rates of nesting (such as 2018) and other years providing suitable conditions, during non‐drought “normal” rainfall conditions that supported an extended nesting season, including 2013 (normal), 2015 (El Niño‐ weak to very strong) and 2019 (weak El Niño to normal). Conditions in the field can vary due to the El Niño/La Niña events, causing a cycle of years with extreme rainfall and extreme drought, which both potentially affect nest success (Bolger et al., 2005; Zuckerberg et al., 2018). Due to the sub‐optimal environmental conditions during the years with the fewest nests, we would expect that these years would have significantly lower nest success than other years. Previous studies on shorebird species have shown varying nest survival responses in years of low nesting. In arctic species, nest survival was not reduced in years with few nests (Smith & Wilson, 2010), whereas a study on tropical Kentish plovers in Cabo Verde found that nest failure was also higher in years with low nesting rates (Engel et al., 2023). Alternatively, individuals that opt to skip breeding in years with more extreme climates might have better success in a following year. In common (*Uria aalge*), the frequency of not breeding was higher in years with higher than average temperatures, although skipping did not improve the breeding success in future years (Reed et al., 2015). In our study, there was no difference in nest survival for years with lower numbers of nesting attempts. However, the factor of year was within supported final models for all three species, suggesting that nest abundance or the environmental conditions that reduced the overall nesting rates, can also affect the nest success of each individual nest to some extent. As year is a combined factor in the final supported models, our results suggest that the effects of year, combined with the life history traits, affect the nest survival.

The effect of El Niño/ La Niña was included in the top models for all Kittlitz's and white‐fronted species plovers, but was not as influential in Madagascar plovers as other factors. Reduced effects of climatic cycles could be an effect of additional parental effort within Madagascar plovers in the incubation of the nest or the defence of nests against predators (Gómez‐Serrano & López‐López, 2016) during El Niño/La Niña events with less optimal environmental conditions. Additional parental effort may mean the parents will expend their own body condition as nesting success remains constant. Weithman et al. (2017) found that adult condition in piping plovers (USA) was negatively correlated with average reproductive output in the recent breeding season, but adult survival was not affected. Alternatively, years with drought‐like conditions may affect the post‐hatching and fledging survival of the chicks rather than the nest survival, such as in the mountain plover (*AA. montanus*, Dinsmore, 2008). However, El Niño/ La Niña variation could become a more prominent effect in the future due to ongoing climate change. The field site at Andavadoaka already experiences high temperatures ranging from 27.3°C in July to 32.2°C in December with average low temperatures ranging from 19.7°C in July to 26.3°C in January (per the Weather Atlas), so potentially behavioural adjustments may not be adequate in the future to ensure a consistent daily nest survival. Temperatures that were too cold in high‐elevation populations (Pierce et al., 2019) and temperatures that were too hot in low‐elevation populations (Dreitz et al., 2012) both negatively affected nest success of mountain plovers. Effects of extreme conditions may not be relevant for the species in this study since El Niño/ La Niña effects were not factors in the final models. Annual conditions can restrict the population in terms of the environmental variation in their nesting ability, but also significantly affect the phenological window during which birds can successfully breed, while also showing that nesting success patterns are not necessarily uniform across a species' distribution (Dreitz et al., 2012; Pierce et al., 2019).

Contrary to our expectations, we found that there was little variation in daily nest survival between the three species, despite the large interspecific differences in nesting behaviours. The similarity in nest survival could suggest that differences in parental care strategies do not cause significant differences in nest success among our species despite in the variation between uni‐ vs. biparental care. However, the species were affected by different factors, with Kittlitz's plovers being less sensitive than white‐fronted and Madagascar plovers to both age of the nest and the year. In arctic‐breeding sanderlings (*Calidris alba*), there was a large difference in nest survival between uniparental and biparental care due to the amount of time that the nest is left unattended. Uniparental pairs had three times the nest recess frequency and a total recess duration seven times higher than that of biparental species (Moreau et al., 2018). In other bird species, a uniparental adult will have a higher daily energy expenditure so their recess time from the nest must include foraging but also must be as short as possible, therefore their nest is left unattended for a longer period of time than biparental parents, with greater exposure to environmental conditions and an increased risk of predation (Leniowski & Węgrzyn, 2018; Moreau et al., 2018; Smith et al., 2012). In Eurasian blackcaps (*Sylvia atricapilla*), biparental parents that display higher synchrony between their off bouts significantly increased their nest survival by decreasing activity at the nest (Leniowski & Węgrzyn, 2018). The risks could be similar within ground‐nesting birds as the predation risks are often associated with visual predators. An increased total duration of recesses and frequency of nest recess can increase predation risk (Leniowski & Węgrzyn, 2018; Meyers et al., 2019; Smith et al., 2012). However, a difference in factors affecting nest survival between parental care strategies was not found among in the three Malagasy species. The lack of difference could be due to the extreme conditions of temperature and rainfall found within the breeding habitat, or potentially, differences in sensitivity that do not lead to reduced nest survival.

Over the 12‐year period of this study, there was no evidence for a long‐term trend for reductions in nest survival for any of the three species studied. Hence, estimates of nest survival are currently stable, although low, for all three species, which is most relevant for the endangered Madagascar plover. Some species may be selected to have longer lifespans to offset low rates of nest survival per year (Murphy, 2007) or alternatively, higher annual productivity with more nesting attempts within a year. However, substantial variation was found that could link the environment experienced by the nests to the overall stochastic climate associated with Madagascar. Due to climate change, Madagascar is expected to become more stochastic with greater variation in the wet and dry years, with both heavier rainfall and more extreme droughts (Abiodun et al., 2019; Masih et al., 2014). Annual variation in nest survival needs to be monitored from a conservation perspective to ensure that suitable mitigation measures are applied before reproductive losses contribute to a decline in the population of the endangered Madagascar plover. For example, predator removal, as the predators in Madagascar are mainly feral dogs, or nest exclosures to reduce human disturbance and predation of newly established nests, where nest survival rates are lower. Monitoring could detect demographic changes and hence highlight when additional mitigation measures are required, especially when concentrating on endemic species which could be more heavily affected by climate change (de Witt, 2003). For example, the wetlands and saltmarsh habitats used for breeding by the plover species are being increasingly encroached on by human activities and disturbance (Kennish, 2001; Lomnicky et al., 2019). In other island populations in similar habitats, such as the Kentish plover in Cape Verde (Engel et al., 2023), nest survival has declined in recent years, hence it is important to ensure demographic losses do not also occur in the endemic populations.

In conclusion, we found that despite differences in social systems, nest success was affected by similar factors among Kittlitz's, white‐fronted and Madagascar plovers breeding in the saltmarsh habitats at Andavadoaka, Madagascar. Our analyses were focused on the incubation stage, and parental care after hatching and renesting ability may have differed among species. Daily nest survival was similar among the three species with year, clutch size and age of nest effects all featuring in the models with the most support. Clutch size was a factor with positive effects on the nest survival within the supported models for all three species, and age of nest also contributed to the most likely models for white‐fronted and Madagascar plovers. In summary, our results suggest that multiple factors play a strong combined role in nest survival and determine nest survival rather than one factor by itself, improving our understanding of population dynamics of wild animals in understudied tropical regions. The nest survival estimates could provide insights into the population decline of the endangered species, Madagascar plover. However, the factors affecting nest survival in this endangered species were not different from other congeneric species in the area. Given the forecasted changes in climatic conditions, the environmental driver of nest survival in the Kittlitz's plovers suggests that extreme climatic variation may contribute more heavily in the future.

## AUTHOR CONTRIBUTIONS

**Claire E. Tanner:** Conceptualization (equal); data curation (equal); formal analysis (equal); investigation (equal); project administration (equal); visualization (equal); writing – original draft (equal); writing – review and editing (equal). **William Jones:** Conceptualization (equal); methodology (equal); writing – review and editing (equal). **Vojtěch Kubelka:** Methodology (equal); writing – review and editing (equal). **Barbara Caspers:** Data curation (equal); funding acquisition (equal); resources (equal). **Oliver Krueger:** Data curation (equal); funding acquisition (equal); resources (equal). **Tafita Jaona Mijoro:** Data curation (equal); methodology (equal). **Brett K. Sandercock:** Investigation (equal); methodology (equal); writing – review and editing (equal). **Sama Zefania:** Conceptualization (equal); data curation (equal); methodology (equal); resources (equal). **Tamás Székely:** Conceptualization (equal); investigation (equal); resources (equal); supervision (equal); writing – original draft (equal); writing – review and editing (equal).

## FUNDING INFORMATION

Fieldwork was supported by ÉLVONAL‐KKP 126949 of NKFIH the National Research, Development and Innovation Office of Hungary (PI: TS): ‘Sex role evolution: testing the impacts of ecology, demography and genes’. The Royal Society Wolfson Merit Award was also awarded to TS (WM170050 to TS). Preparation of the manuscript was supported by basic funding to the Norwegian Institute for Nature Research from the Research Council of Norway (Project No. 160022/F40) and by the Czech Science Foundation (Junior Star GAČR project 31‐2307692M_Kubelka).

## CONFLICT OF INTEREST STATEMENT

The authors have no conflicts of interest to declare.

## Supporting information


Appendix S1.

## Data Availability

Data from this field study are available at DataDryad via https://datadryad.org/stash/dataset/doi:10.5061/dryad.41ns1rnmt
