# Supplementary material for: Variation in nest survival of three species of tropical plovers in Madagascar with clutch size, age of nest, year and El Niño effect
Source: Ecol Evol. 2024 Sep 16;14(9):e70269. doi: 10.1002/ece3.70269 (PMC11405060; doi:10.1002/ece3.70269)
Supplement: Supplementary file 1 — Appendix S1. [file ECE3-14-e70269-s001.docx]

**Supplementary Information:**


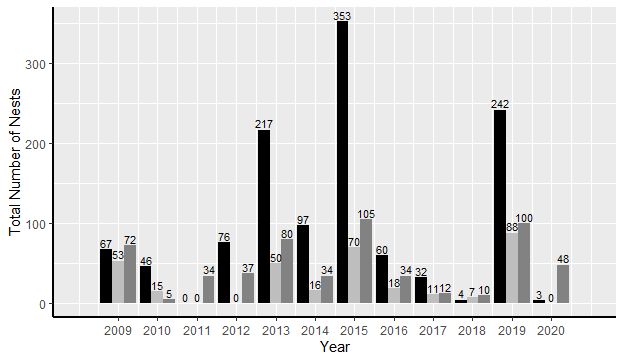


Supplementary Figure 1: Total number of nests found per year for three species of tropical plovers at Andavadoaka, Madagascar, 2009-2020 (sample size of nests above each bar). Kittlitz’s plovers (KiP) in black, Madagascar plovers (MP) in light grey and white-fronted plovers (WfP) in dark grey.

**Effort Effects**

Field effort was measured as the total number of person days per year. Field effort had a positive correlation with the number of nests found in white-fronted plovers and Madagascar plovers, but no correlation in Kittlitz’s plovers (KiP, r(10) = 0.539, p = 0.071; MP, r(10) = 0.689, p = 0.013; WfP, r(10) = 0.619, p = 0.032). However, the percentage of nests which hatched had no correlation with personnel effort (KiP, r(10) = 0.049, p = 0.879; MP, r(10) = 0.245, p = 0.443; WfP, r(10) = 0.008, p = 0.980), in all of the three species (Supplementary Figure 1).


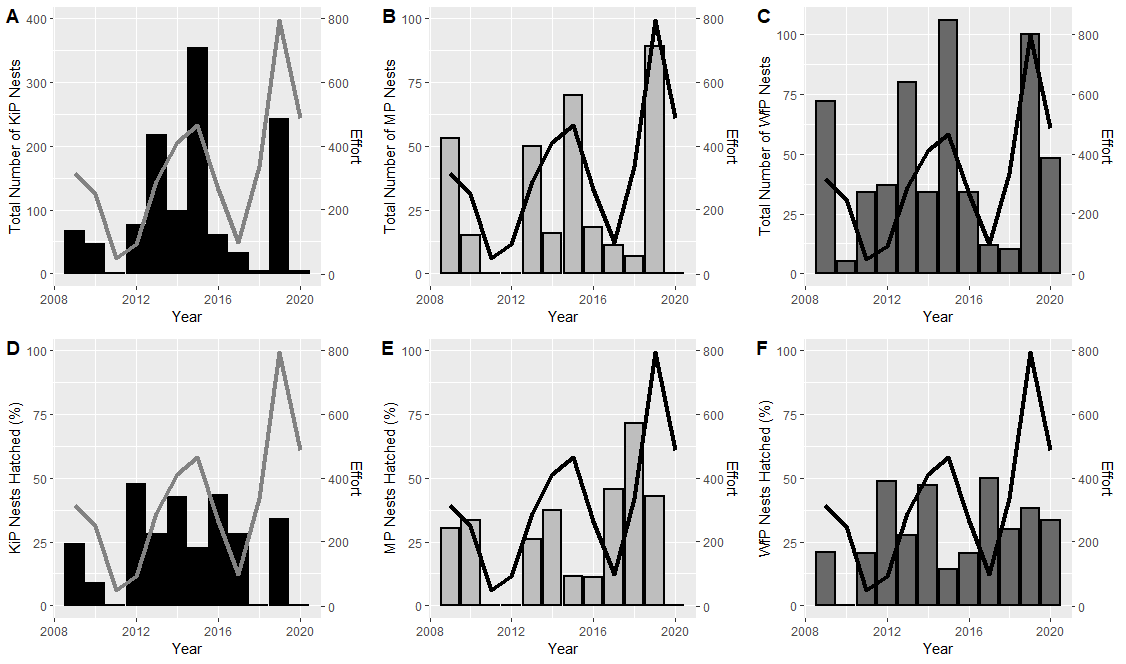


Supplementary Figure 2: Comparison between the human effort days per year (shown by the solid line in every panel of the graph) versus the number of nests found each year (A, B, C) per species, and versus the percentage of hatched nests each year (D, E, F) per species at Andavodoaka, Madagascar, 2009-2020. Kittlitz’s plovers (KiP) in black, Madagascar plovers (MP) in light grey and white-fronted plovers (WfP) in dark grey.

Supplementary Table 1: Fates of nests (% of total nests) for Kittlitz’s, White-fronted and Madagascar plovers. Other losses include nests failed due to flooding, trampling and nests with infertile eggs.

|  | **Hatched (% ± SE)** | **Depredated (% ± SE)** | **Abandoned by parents (% ± SE)** | **Other**  **(% ± SE)** | **Unknown fate**  **(% ± SE)** | **Sample size (N)** |
| --- | --- | --- | --- | --- | --- | --- |
| **Kittlitz’s** | 29.7 ±0.02 | 48.3 ±0.02 | 6.8 ±0.03 | 1.1 ±0.03 | 13.9 ±0.03 | 1185 |
| **Madagascar** | 29.8 ±0.04 | 44.1 ±0.03 | 2.4 ±0.05 | 1.5 ±0.06 | 22.2 ±0.04 | 327 |
| **White-fronted** | 28.5 ±0.03 | 44.4 ±0.02 | 4.4 ±0.04 | 2.8 ±0.04 | 19.9 ±0.03 | 565 |

Supplementary Table 2: Calibrated egg stage of the three plovers (KiP = Kittlitz’s plover, MP=Madagascar plover and WfP=White-fronted plover). The duration of incubation from completion of laying to hatching was 25.8 days ± 0.3 SE for Kittlitz’s, 28.6 days ± 0.3 SE for Madagascar, and 28.5 days ± 2.3 SE for White-fronted plovers.

| **KiP** Incubation stage | A and laying | B | C | D | E | F | Hatch |
| --- | --- | --- | --- | --- | --- | --- | --- |
| Number of eggs used for calibration | 54 | 15 | 13 | 38 | 54 | 20 | 26 |
| Number of days incubated (mean ± SE) | 2.3 ± 0.1 | 3.3 ± 0.3 | 7.2 ± 0.5 | 12.2 ± 0.3 | 20.3 ± 0.5 | 25.0 ± 0.4 | 25.8 ± 0.3 |
| Number of days incubated (mean ± sd) | 2.3 ± 1.0 | 3.3 ± 1.2 | 7.2 ± 1.6 | 12.2 ± 1.8 | 20.3 ± 3.8 | 25.0 ± 1.7 | 25.8 ± 1.5 |
| **MP** Incubation stage | A and laying | B | C | D | E | F | Hatch |
| Number of eggs used for calibration | 50 | 6 | 8 | 24 | 36 | 2 | 13 |
| Number of days incubated (mean ± SE) | 2.3 ± 0.1 | 3.0 ± 0.4 | 6.6 ± 0.5 | 13.1 ± 0.5 | 20.3 ± 0.7 | 27.0 ± 1.0 | 28.6 ± 0.3 |
| Number of days incubated (mean ± sd) | 2.3 ± 1.1 | 3.0 ± 1.1 | 6.6 ± 1.3 | 13.1 ± 2.4 | 20.3 ± 4.2 | 27.0 ± 1.4 | 28.6 ± 1.2 |
| **WfP** Incubation stage | A and laying | B | C | D | E |  | Hatch |
| Number of eggs used for calibration | 80 | 2 | 2 | 2 | 10 |  | 4 |
| Number of days incubated (mean ± SE) | 2.5 ± 0.2 | 9.5 ± 1.5 | 13.0 ± 2.0 | 11.0 ± 2.0 | 19.3 ± 1.1 |  | 28.5 ± 2.3 |
| Number of days incubated (mean ± sd) | 2.5 ± 1.5 | 9.5 ± 2.1 | 13.2 ± 2.8 | 11.0 ± 2.8 | 19.3 ± 3.4 |  | 28.5 ± 4.7 |

Few samples were collected at the stages B, C, D, and E of WfP, and there was no sample at the stage F.

The stages were based on floatation angle of the longitudinal horizontal axis of the egg; A = egg laying on the bottom of the dish, B = < 45 degrees , C = 45 < 90 degrees, D = 90 degrees, E = egg floats with the blunt end pointing down, F = blunt edge of the egg appears on the surface of the water.

Supplementary Table 3: Age of nests at first discovery in 6 different age categories, using floatation methods, for Kittlitz’s, Madagascar and white-fronted plovers.

| **Species \| Nest Age (days)** | **≤ 5** | **5 ≤ 10** | **10 ≤ 15** | **15 ≤ 20** | **20 ≤ 25** | **25 ≤ Hatch** |
| --- | --- | --- | --- | --- | --- | --- |
| **Kittlitz’s** | 623 (52.6%) | 207 (17.5%) | 129 (10.9%) | 9 (0.8%) | 216 (18.2%) | 1  (0.0%) |
| **Madagascar** | 179 (54.6%) | 45 (13.7%) | 34  (10.4%) | 3 (0.9%) | 52 (15.9%) | 14  (4.3%) |
| **White-fronted** | 90 (17.5%) | 95 (18.5%) | 189 (36.8%) | 101 (19.7%) | 36 (7.0%) | 2  (0.4%) |
